# A simple vapor-diffusion method enables protein crystallization inside the HARE serial crystallography chip

**DOI:** 10.1107/S2059798321003855

**Published:** 2021-05-19

**Authors:** Brenna Norton-Baker, Pedram Mehrabi, Juliane Boger, Robert Schönherr, David von Stetten, Hendrik Schikora, Ashley O. Kwok, Rachel W. Martin, R. J. Dwayne Miller, Lars Redecke, Eike C. Schulz

**Affiliations:** a Department for Atomically Resolved Dynamics, Max-Planck-Institute for Structure and Dynamics of Matter, Luruper Chaussee 149, 22761 Hamburg, Germany; b Department of Chemistry, University of California, Irvine, CA 92697-2025, USA; c Hamburg Centre for Ultrafast Imaging, Universität Hamburg, HARBOR, Luruper Chaussee 149, 22761 Hamburg, Germany; d Institute of Biochemistry, Center for Structural and Cell Biology in Medicine, University of Lübeck, Ratzeburger Allee 160, 23562 Lübeck, Germany; e Photon Science, Deutsches Elektronen-Synchrotron (DESY), Notkestrasse 85, 22607 Hamburg, Germany; f European Molecular Biology Laboratory, Hamburg Unit c/o Deutsches Elektronen-Synchrotron, 22607 Hamburg, Germany; g Scientific Support Unit Machine Physics, Max-Planck-Institute for Structure and Dynamics of Matter, Luruper Chaussee 149, 22761 Hamburg, Germany; h Department of Molecular Biology and Biochemistry, University of California, Irvine, CA 92697-3900, USA; i Department of Physics, Universität Hamburg, Jungiusstrasse 9, 20355 Hamburg, Germany; j Departments of Chemistry and Physics, University of Toronto, 80 St George Street, Toronto, ON M5S 3H6, Canada

**Keywords:** fixed-target crystallography, serial crystallography, protein crystallization, *in cellulo* crystallization, *in vivo* crystals

## Abstract

Fixed-target serial crystallography has become an important method for the study of protein structure and dynamics at synchrotrons and X-ray free-electron lasers. However, sample homogeneity, consumption and the physical stress on samples remain major challenges for these high-throughput experiments, which depend on high-quality protein microcrystals. The batch crystallization procedures that are typically applied require time- and sample-intensive screening and optimization. Here, a simple protein crystallization method inside the features of the HARE serial crystallography chips is reported that circumvents batch crystallization and allows the direct transfer of canonical vapor-diffusion conditions to in-chip crystallization. Based on conventional hanging-drop vapor-diffusion experiments, the crystallization solution is distributed into the wells of the HARE chip and equilibrated against a reservoir with mother liquor. Using this simple method, high-quality microcrystals were generated with sufficient density for the structure determination of four different proteins. A new protein variant was crystallized using the protein concentrations encountered during canonical crystallization experiments, enabling structure determination from ∼55 µg of protein. Additionally, structure determination from intracellular crystals grown in insect cells cultured directly in the features of the HARE chips is demonstrated. *In cellulo* crystallization represents a comparatively un­explored space in crystallization, especially for proteins that are resistant to crystallization using conventional techniques, and eliminates any need for laborious protein purification. This in-chip technique avoids harvesting the sensitive crystals or any further physical handling of the crystal-containing cells. These proof-of-principle experiments indicate the potential of this method to become a simple alternative to batch crystallization approaches and also as a convenient extension to canonical crystallization screens.

## Abstract 2

The in-chip crystallization and structure determination of four different soluble proteins and an intracellular protein using serial synchrotron crystallography is reported.

## 1. Introduction   

X-ray crystallography has contributed dramatically to our current understanding of biomolecular processes. The obtained protein structures, however, only capture one static moment in a dynamic system as most data are collected at cryogenic temperatures. While this technique limits radiation damage, experiments on nonfrozen samples with native ligands are essential for a deeper understanding of protein structural dynamics and chemical activity. Exploiting this opportunity, recent developments in serial crystallography now allow the study of structural changes during a reaction cycle (Chapman, 2019 ▸). In serial crystallography, diffraction patterns are collected from many crystals, often thousands, and data are indexed and merged for protein structure solution (Chapman *et al.*, 2011 ▸). These developments have been triggered by increased source brightness at X-ray free-electron lasers (XFELs) and third/fourth-generation synchrotrons, as well as advances in beam micro-focusing, allowing the X-ray exposure time per crystal to be drastically reduced and experiments to be carried out on smaller crystals. With serial data-collection strategies, dose accumulation is mitigated and room-temperature measurements become viable. In addition to time-resolved applications, serial crystallography promises to be particularly useful for systems for which the growth of a large single crystal remains difficult, such as many membrane proteins or intracellularly grown protein crystals (Redecke *et al.*, 2013 ▸; Nass *et al.*, 2020 ▸). Serial data collection has enabled significant developments in time-resolved crystallography using both XFELs (Tenboer *et al.*, 2014 ▸; Barends *et al.*, 2015 ▸; Nango *et al.*, 2016 ▸; Pande *et al.*, 2016 ▸) and synchrotron sources (Schulz *et al.*, 2018 ▸; Mehrabi, Schulz, Dsouza *et al.*, 2019 ▸; Mehrabi, Schulz, Agthe *et al.*, 2019 ▸; Weinert *et al.*, 2019 ▸), as these typically require data collection from nonfrozen samples. Additionally, crystals in the micrometre size regime are required for more homogeneous reaction initiation throughout the crystal for both optical excitation and *in situ* mixing.

The need for rapid exchange of crystals in the beam path, in order to collect thousands of diffraction images in a practical time frame, has advanced new methods of sample delivery, including both injection-based and fixed-target approaches (Grünbein & Nass Kovacs, 2019 ▸; Martiel *et al.*, 2019 ▸). For the ultrafast timescales accessible with XFELs, injection methods have been used with the most success (Boutet *et al.*, 2012 ▸; Chapman *et al.*, 2011 ▸; Tenboer *et al.*, 2014 ▸; Chapman, 2019 ▸). However, fixed-target sample delivery offers the distinct advantages of comparably low sample consumption, high hit rates and access to longer and more versatile time delays (Hunter *et al.*, 2015 ▸; Oghbaey *et al.*, 2016 ▸; Davy *et al.*, 2019 ▸; Tolstikova *et al.*, 2019 ▸; Owen *et al.*, 2017 ▸; Sherrell *et al.*, 2015 ▸; Schulz *et al.*, 2018 ▸). Moreover, fixed-target serial femtosecond crystallography (FT-SFX) is ideally suited for efficient *in cellulo* diffraction data collection using living, crystal-containing cells, as recently demonstrated by Lahey-Rudolph *et al.* (2021 ▸). Crystallization of recombinant proteins in living cells is an exciting new approach that is particularly important for proteins that are not accessible for crystallization using established *in vitro* screening strategies, as shown for *Trypano­soma brucei* IMPDH and fully glycosylated *T. brucei* CatB (Redecke *et al.*, 2013 ▸; Nass *et al.*, 2020 ▸). High-resolution structural information on several recombinant proteins has already been obtained from the diffraction of *in cellulo* crystals (Schönherr *et al.*, 2018 ▸).

In this study, we use the previously described HARE (‘Hit-And-REturn’) chip design that has precisely defined crystal locations. These lithographically fabricated silicon chips hold the microcrystals in random orientations in inverted pyramidal bottomless wells, also called features (Mehrabi *et al.*, 2020 ▸; Oghbaey *et al.*, 2016 ▸; Davy *et al.*, 2019 ▸). The HARE chips are mountable on high-speed translation stages that enable rapid exchange of crystals through the beam path, giving routine data-collection rates of ∼30 Hz, although higher rates of up to ∼100 Hz have been achieved with these stages (Schulz *et al.*, 2018 ▸; Sherrell *et al.*, 2015 ▸). The HARE-chip design is integral to efficient time-resolved data collection via the ‘hit-and-return’ (HARE) method, in which the time delays are mechanically set through movement of the stages to precise crystal positions. This enables efficient data collection for short (milliseconds) and long (seconds to minutes) time delays (Schulz *et al.*, 2018 ▸; Mehrabi *et al.*, 2020 ▸). Reaction initiation via optical excitation as well as *in situ* mixing have been demonstrated for reactions on millisecond to minute timescales (Schulz *et al.*, 2018 ▸; Mehrabi, Schulz, Dsouza *et al.*, 2019 ▸; Mehrabi, Schulz, Agthe *et al.*, 2019 ▸).

In general, the challenge of efficient crystal loading remains for fixed-target approaches. In previous studies using the HARE-chip design, the proteins were first crystallized in batch and the crystal slurry was subsequently transferred to the chip (Schulz *et al.*, 2017 ▸, 2018 ▸; Lučić *et al.*, 2020 ▸; Moreno-Chicano *et al.*, 2019 ▸; Ebrahim, Appleby *et al.*, 2019 ▸; Ebrahim, Moreno-Chicano *et al.*, 2019 ▸; Mehrabi, Schulz, Dsouza *et al.*, 2019 ▸; Davy *et al.*, 2019 ▸; Owen *et al.*, 2017 ▸; Mehrabi *et al.*, 2020 ▸). Although the generation of microcrystals based on canonical vapor-diffusion conditions is greatly simplified by our vacuum-crystallization procedure, large-scale batch crystallization can still be challenging, including potentially time- and protein-consuming screening and optimization (Beale *et al.*, 2019 ▸; Martin & Zilm, 2003 ▸; Stohrer *et al.*, 2021 ▸). Recently, on-chip crystallization has been demonstrated for the Roadrunner chip. However, because the features lack concavity, the crystals are distributed in random locations on the surface of the chips. This preparation is therefore not easily adaptable to the HARE data-collection method for time-resolved applications, as the time delays are achieved by the periodic spacing of the crystals. Additionally, the high-speed movement of the stages might cause shifting of the crystal positions on a flat surface, impeding the return to a particular crystal at a set time delay (Lieske *et al.*, 2019 ▸; Schulz *et al.*, 2018 ▸; Mehrabi *et al.*, 2020 ▸).

To overcome these challenges, we sought a solution that would (i) further reduce the amount of protein sample required, (ii) minimize the physical stress on the crystals, while maintaining compatibility with time-resolved data collection via the HARE method, and (iii) streamline the transition from a canonical vapor-diffusion crystallization condition to a microcrystallization condition suitable for serial crystallo­graphy.

Here, we demonstrate an *in situ* crystallization method in which the crystals are directly grown within a <1 nl volume of the HARE-chip wells. This method eliminates virtually all crystal handling and also drastically reduces protein consumption. Initially, three model systems were crystallized inside the features of the chip: lysozyme, proteinase K and xylose isomerase. To test this method with a challenging crystallization target, we used a novel variant of the human eye lens protein γS-crystallin, from a class of proteins which are notoriously difficult to crystallize. To further emphasize the reduction of physical stress on the microcrystals, *in situ* crystallization was explored with the fungal protein HEX-1, microcrystals of which grow in insect cells directly cultured on the chips. The HARE chips enabled fast and efficient diffraction data collection from *in cellulo* protein crystals in living cells at a synchrotron source. Room-temperature, serial X-ray crystallography structures were successfully determined for all five systems.

## 2. Materials and methods   

### 2.1. Protein preparation and crystallization   

#### 2.1.1. Lysozyme   

Lysozyme from chicken egg white was purchased from Sigma (catalogue No. L6876). The lyophilized powder was dissolved to 65 mg ml^−1^ in 50 m*M* sodium acetate pH 4.7. 5 µl protein solution was mixed with 5 µl precipitant solution (33% PEG 4000, 0.5 *M* NaCl, 50 m*M* sodium acetate pH 4.5) prior to deposition on the chip.

#### 2.1.2. Proteinase K   

Proteinase K from *Tritirachium album* was purchased from Sigma (catalogue No. P6556). The lyophilized powder was dissolved to 60 mg ml^−1^ in 50 m*M* HEPES pH 7.0. 5 µl protein solution was mixed with 5 µl precipitant solution (1 *M* NaNO_3_, 0.1 *M* sodium citrate pH 6.5) prior to deposition on the chip.

#### 2.1.3. Xylose isomerase   

Xylose isomerase from *Streptomyces rubiginosus* was purchased from Hampton Research (catalogue No. HR7-102) and stored for several years. The stock crystals were dissolved in water, washed repeatedly in a concentrator to exchange the storage buffer and concentrated to 80 mg ml^−1^. 5 µl protein solution was mixed with 5 µl precipitant solution [35%(*w*/*v*) PEG 3350, 200 m*M* lithium sulfate, 10 m*M* HEPES/NaOH pH 7.5] prior to deposition on the chip.

#### 2.1.4. Human γS-crystallin variant   

The human γS-crystallin variant (N15D/Q121E/N144D/N54D/Q93E/Q64E/Q17E/Q107E/Q71E) was created using site-directed mutagenesis PCR from the wild-type construct with an N-terminal 6×His tag and a Tobacco etch virus (TEV) protease cleavage sequence. *Escherichia coli* Rosetta (DE3) cells were transformed with a pET-28a(+) vector (Novagen, Darmstadt, Germany) containing the human γS-crystallin variant cDNA. Overexpression was achieved using Studier’s autoinduction protocol (Studier, 2005 ▸) for 1 h at 37°C followed by 20–24 h at 25°C. The cells were lysed via sonication and the supernatant of the lysate was loaded onto an Ni–NTA column (Applied Biosystems, Foster City, California, USA). The tagged protein was eluted using imidazole and the tag was cleaved by TEV protease (produced in-house). The protein and tag were separated by reapplication to the Ni–NTA column. A final size-exclusion purification was performed on a HiLoad 16/600 Superdex 75 pg column (GE Healthcare Life Sciences, Piscataway, New Jersey, USA). The protein was lyophilized for storage, resolubilized, buffer-exchanged into 50 m*M* HEPES pH 7.0, 5 m*M* DTT, concentrated to 20 mg ml^−1^ and stored at 4°C for several months. 5 µl protein solution, diluted to 11 mg ml^−1^ in 50 m*M* HEPES pH 7.0, was mixed with 5 µl precipitant solution [20%(*w*/*v*) PEG 3350, 0.1 *M* sodium acetate pH 5.45] prior to deposition on the chip.

#### 2.1.5. HEX-1   

Cloning of the HEX-1 gene (GenBank accession No. XM_958614) from the filamentous fungus *Neurospora crassa* has been described previously (Lahey-Rudolph *et al.*, 2020 ▸). In brief, the HEX-1 coding sequence was amplified using the primers 5′-TACTACGACGACGACGXTCACG-3′ (sense) and 5′-GAGGCGGGAACCGTGGACG-3′ (antisense) and blunt end-ligated into the EheI site of a pFastBac1 vector containing the sequence 5′-ATGGGCGCCTAA-3′ between the BamHI and HindIII restriction sites. For baculovirus production, recombinant bacmid DNA was generated in *E. coli* DH10EmBacY cells (Geneva Biotech) and used for lipofection of *Spodoptera frugiperda* Sf9 insect cells. The virus titer was calculated using the TCID_50_ (tissue-culture infectious dose) in a serial dilution assay as described previously (Lahey-Rudolph *et al.*, 2020 ▸).

Intracellular crystallization of HEX-1 was achieved by infecting 9 × 10^5^ *Trichoplusia ni* High Five insect cells with the Hex-1-encoding recombinant baculovirus (rBV) in a well of a six-well cell-culture plate in 2 ml serum-free ESF921 medium (Expression Systems) with a multiplicity of infection (MOI) of 1. The cells were incubated at 27°C for 96 h. *In cellulo* crystal formation was verified by light microscopy using a Nikon Ts2R-FL microscope employing differential interference contrast. For chip loading, the cells were resuspended in cell-culture medium in the well and transferred into a 1.5 ml tube. After centrifugation for 1 min at 200*g*, the cell pellet was resuspended in 200 µl ESF921 medium and the cell suspension was pipetted onto the chip. Excess medium was removed using a custom-made vacuum system (Mehrabi *et al.*, 2020 ▸).

### 2.2. *In situ* protein crystallization   

#### 2.2.1. HARE-chip design   

**Figure 1:**
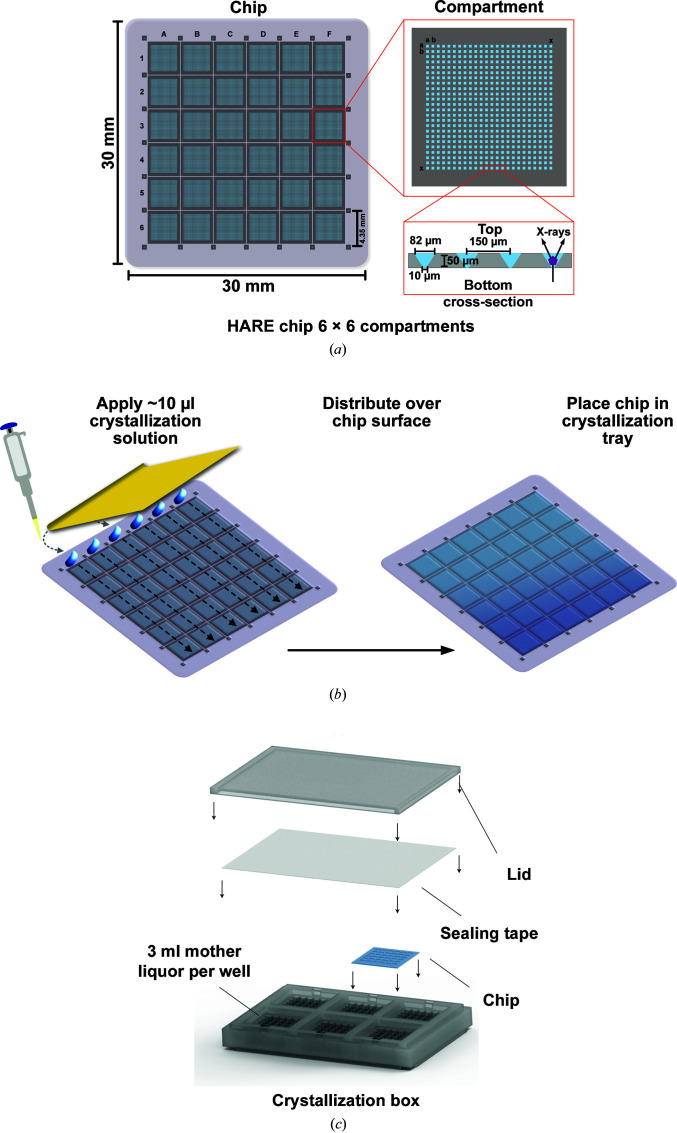
Vapor-diffusion micro-crystallization procedure within the HARE chip. (*a*) Silicon HARE chip with 6 × 6 compartments containing 24 × 24 features per compartment. The cross section shows the dimensions of the features. (*b*) A blade is used to spread a minimal amount of crystallization mixture evenly over the surface of the chip and into the features. (*c*) Six chips can be placed in the crystallization tray to conduct canonical vapor-diffusion crystallization experiments. Sample consumption is minimized to 10 µl crystallization solution (using just 5 µl of protein stock solution) per chip.

The HARE chips used in these experiments have previously been described in detail (Mehrabi *et al.*, 2020 ▸). Briefly, lithographic techniques were used to produce >20 000 tapered bottomless wells, also called features, in single-crystal silicon. These have 6 × 6 or 8 × 8 compartments containing 24 × 24 or 20 × 20 features, respectively, with top openings of 82 × 82 µm that taper to 10 × 10 or 15 × 15 µm (Fig. 1 ▸ *a*). Chips with larger bottom openings of 25 × 25 µm were used for the experiments with intracellular crystals and in order to visualize the crystals in a stereomicroscope. These previously undescribed chips had 3 × 3 compartments with 53 × 53 features.

#### 2.2.2. In-chip crystallization   

10 µl of the premixed solution containing equal amounts of protein and precipitant solutions was deposited via pipette in even droplets across one row of compartments of the freshly glow-discharged chips. A thin, flexible metal blade was used to spread the liquid evenly over the surface and into the features of the chip (Fig. 1 ▸ *b*). The chip was quickly sealed with transparent tape inside the custom chip crystallization tray over 3 ml precipitant solution. Growth was observed with an Olympus SZX10 microscope and images were captured with an Olympus DP27 camera. The *Olympus Stream* software was used for size measurements.

#### 2.2.3. Vapor-diffusion micro-crystallization tray   

To streamline vapor-diffusion micro-crystallization with the HARE chips, we designed a custom in-chip crystallization box. Following a conventional vapor-diffusion approach, the crystallization solution can be loaded onto the chip and placed on the crystallization box filled with mother liquor. Sealing of the box with conventional crystallization tape prevents dehydration of the mother liquor and allows in-chip crystallization of the proteins. This box roughly matches the SBS plate format, and several boxes can be stacked for convenient storage (Fig. 1 ▸ *c*). Information to reproduce the crystallization tray using a 3D printer is available in the supporting information. All 3D-printed parts were printed from Formlabs clear resin FLGPCL04 (Somerville, Massachusetts, USA).

#### 2.2.4. *In cellulo*, in-chip crystallization   

For in-chip crystallization of HEX-1 in living insect cells, the chips were coated with a 0.1 mg ml^−1^ aqueous solution of poly-d-lysine for 1 h and washed twice with PBS pH 7.0. The chips were subsequently submerged in ESF921 medium and covered with 9 × 10^5^ High Five cells per chip. After 20 min incubation at 27°C for adhesion of the cells to the surface of the chips, the cells were infected with the HEX-1-encoding rBV at an MOI of 1 and incubated at 27°C until the beamtime at 96 h post-infection (h.p.i). The chips were imaged via the beamline camera using an IR light source. Since the HARE chips are transparent to IR light, the cells can be seen within the feature of the chip as well as on its surface.

#### 2.2.5. Centrifugation for crystal centering into features   

The chips were transferred into a new crystallization tray to avoid spillover of the reservoir solution during centrifugation. They were quickly resealed over ∼500 µl water to maintain hydration. For sensitive crystals, it may be advisable to use mother liquor rather than water. A Concentrator Plus centrifugal evaporator unit (Eppendorf) with an A-2-VC plate rotor was used in centrifuge mode at 1400 min^−1^ without vacuum and the plates were spun for 1 min and returned to their original tray for storage until data collection.

### 2.3. Serial X-ray diffraction experiments   

#### 2.3.1. Serial data collection   

All serial X-ray diffraction experiments were conducted at room temperature on EMBL beamline P14-2 (T-REXX) at the PETRA III synchrotron at DESY, Hamburg (https://www.embl-hamburg.de/services/mx/P14_EH2/). The chips were mounted in chip holders inside a humidified chamber to prevent dehydration (Mehrabi *et al.*, 2020 ▸). They were sealed with 2.5 µm Mylar foil seals and then connected to the SmarAct translation stages as described previously (Schulz *et al.*, 2018 ▸; Sherrell *et al.*, 2015 ▸; Mehrabi *et al.*, 2020 ▸). These three-axis piezo translation stages precisely control the movement of the chip’s features into the X-ray beam. Diffraction images were recorded on an EIGER 4M or a PILATUS 2M detector. Data collection was performed at room temperature at a photon energy of 12.65 or 12.7 keV, with a single diffraction pattern recorded for each feature. Diffraction images were visualized via the *Adxv* software (Arvai, 2019 ▸).

#### 2.3.2. Structure determination   

Diffraction data were processed using *CrystFEL* for peak finding, integration (White *et al.*, 2012 ▸). Phasing was performed by molecular replacement using *Phaser* (McCoy *et al.*, 2007 ▸). PDB entries 1dpx (Weiss *et al.*, 2000 ▸), 6j43 (S. J. Lee & J. Park, unpublished work), 6qni (Mehrabi, Shulz, Agthe *et al.*, 2019 ▸), 6fd8 (Thorn *et al.*, 2019 ▸) and 1khi (Yuan *et al.*, 2003 ▸) were used as search models for lysozyme, proteinase K, xylose isomerase, the γS-crystallin variant and HEX-1, respectively. *Phenix* was used for refinement and the structures were manually edited in *Coot* between rounds of refinement (Liebschner *et al.*, 2019 ▸; Emsley *et al.*, 2010 ▸). Structure images were produced in *PyMOL* (Schrödinger).

## 3. Results   

### 3.1. Proof of principle   

#### 3.1.1. In-chip protein crystallization procedure   

**Figure 2:**
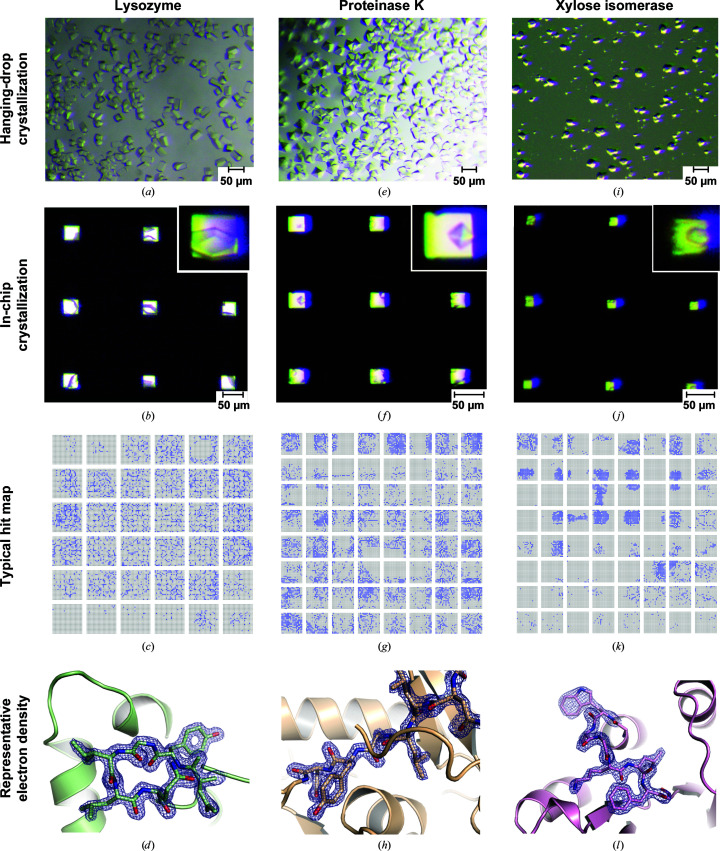
Vapor-diffusion in-chip crystallization of model proteins. In-chip crystallization of the model proteins lysozyme (*a–d*), proteinase K (*e*–*h*) and xylose isomerase (*i*–*l*). Top rows: conventional hanging drops are compared with in-chip crystallization using the same crystallization conditions. The inset shows enlarged images of crystals within the features of the HARE chip. The scale bars represent 50 µm. Third row: a representative hit map is shown for each model protein, where blue indicates a recorded diffraction pattern that was successfully indexed by *CrystFEL*. Bottom row: representative section of the 2*F*_o_ − *F*_c_ electron-density map (contoured at 2σ) after refinement of data from a single chip.

In an initial proof-of-principle experiment, we aimed to demonstrate that proteins can be crystallized directly in the features of the HARE chip using a canonical vapor-diffusion approach (Fig. 1 ▸ *c*). To this end, crystallization conditions were first optimized for lysozyme via traditional hanging-drop vapor-diffusion screens, targeting high nucleation rates and crystals of approximately 20 µm in size in all dimensions (Fig. 2 ▸ *a*). Frequent nucleation was achieved by increasing the protein concentration in the crystallization solution. Further strategies for identifying promising conditions for serial crystallography and optimizing them for nucleation rate and homogeneity have been described by Beale *et al.* (2019 ▸).

Using these optimized conditions, a solution with the same ratio of lysozyme:precipitant concentration was prepared to afford 10 µl crystallization solution. This solution was applied to the top row of the HARE chip and spread across the surface and into the features of the chip with a thin flexible metal blade. Therefore, 5 µl of protein solution, assuming a 1:1 ratio of protein:precipitant, is sufficient to fill a complete chip (Fig. 1 ▸ *b*).

For lysozyme, the amount of protein consumed to fill a single chip was 350 µg. The total volume to fill the chip features can be further decreased to ∼7 µl; however, with lower volumes manual distribution to all corners of the chip becomes more difficult. Additionally, application within a humidity-controlled environment is recommended to avoid rapid evaporation; a home-built solution has previously been described by Mehrabi *et al.* (2020 ▸). The chips were then sealed using standard crystallization sealing tape inside custom-designed crystallization trays (Fig. 1 ▸ *c*). The trays are designed to hold six chips and 3 ml reservoir solution per chip well, to enable either screening of multiple conditions or efficient in-chip crystallization using an established condition. The trays are translucent, allowing observation of the crystals in the features with a stereomicroscope. We anticipate that UV microscopy would be ideally suited to visualize the crystals in the features of the chips and facilitate monitoring of crystallization success; however, as the trays are not UV-transparent, the chips would need to be removed and sealed with transparent foil for evaluation by UV microscopy. In the absence of an appropriate UV microscope, visible-light microscopy was used to analyze in-chip crystallizations prior to data collection. To better visualize the crystals within the features, the conditions for the crystallization solution were first tested on chips with features with 25 × 25 µm bottom openings, as these allow more transmitted light (Fig. 2 ▸ *b*). For diffraction data collection, we used chips with bottom openings of 10–15 µm. The chips were mounted onto the high-speed translation stages for serial data collection on EMBL beamline P14-2 (T-REXX) at PETRA III (Table 1 ▸, Fig. 2 ▸ *c*).

#### 3.1.2. Crystal centering increases hit rates   

**Figure 3:**
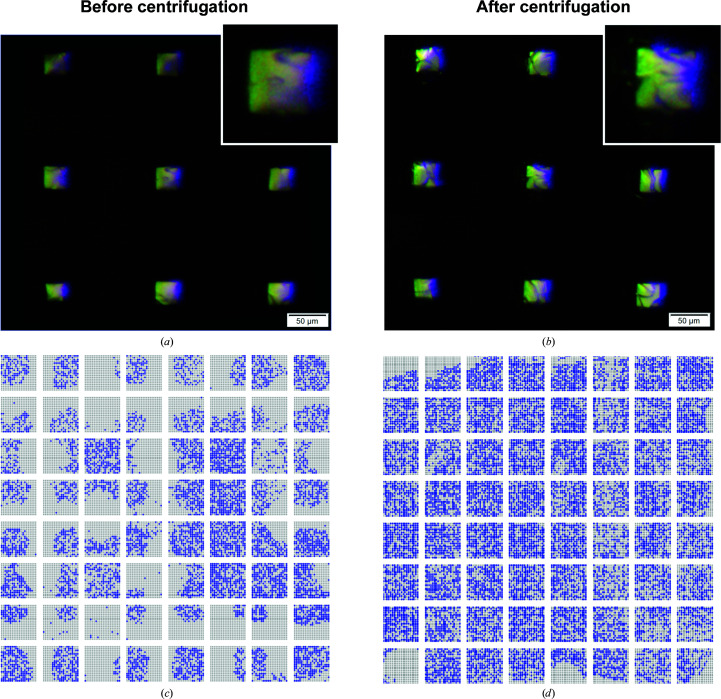
Crystal centering using gentle centrifugation. The same features of a chip with lysozyme crystals are shown (*a*) before and (*b*) after centrifugation for 1 min. Hit maps for two identical chips prepared from the same stock solution of lysozyme:precipitant mixture (*c*) without centrifugation and (*d*) with centrifugation. Blue indicates a recorded diffraction pattern that was successfully indexed by *CrystFEL*.

From visual analysis of the initial lysozyme trials (chips 1 and 2) it was noted that almost all features contained one or more crystals. Initially, however, the hit rates during diffraction data collection were significantly lower than visual inspection would suggest. Presumably, crystals located at the periphery of the features were likely to fall outside the beam path, as the beam [15 × 10 µm (*H* × *V*)] is centered at the openings (10 × 10 µm) of the features. To address this problem, we sought a crystal-centering method. To this end, a post-crystallization centrifugation step was included in the workflow to gently sediment the crystals to the bottom center of the features. The centering of crystals in the features is apparent in the images of the same features of a chip with in-chip-grown lysozyme crystals shown before and after centrifugation. In Fig. 3 ▸(*a*), only the edges of the lysozyme crystals are apparent as they are located at the periphery of the features. After centrifugation, in Fig. 3 ▸(*b*), multiple crystals can be seen in each well and are now likely to be within the beam path. To minimize crystallization variance, two identical lysozyme chips (chips 3 and 4) were then prepared from the same stock solution of protein:precipitant mixture. Chip 3 was used as before, while chip 4 was centrifuged at 1400 rev min^−1^ (∼120*g*) for 1 min. Centrifugation yields up to more than a twofold increase in the indexing rate and does not appear to have detrimental effects on diffraction quality (Table 1 ▸, Figs. 3 ▸ *c* and 3 ▸ *d*). Lysozyme structures were solved at a resolution of 1.70 Å (Fig. 2 ▸ *d*) for chip 3 (not centrifuged) and chip 4 (centrifuged) data sets from in-chip-grown crystals (Table 1 ▸) and the refined structures have been deposited in the Protein Data Bank (PDB entries 7nkf and 7njf, respectively).

#### 3.1.3. Additional model systems can be crystallized inside HARE chips   

In-chip crystallization was then tested on two additional systems with well known crystallization protocols: proteinase K and xylose isomerase. Similar to the strategy for lysozyme, conditions for abundant nucleation and ∼20 µm crystal size were optimized in hanging-drop crystallization setups (Figs. 2 ▸ *e* and 2 ▸ *i*). The optimized conditions were then directly applied to the chip in droplets with a total of 10 µl (5 µl protein solution mixed with 5 µl mother liquor) and spread into the chip features, yielding in-chip-grown crystals (Figs. 2 ▸ *f* and 2 ▸ *j*). The total amount of protein sample consumed for each chip for proteinase K and xylose isomerase was 300 and 400 µg, respectively. Data were collected from six proteinase K chips and one xylose isomerase chip. Using data sets from proteinase K chip 3 and xylose isomerase chip 1, structures were solved at resolutions of 1.65 and 1.90 Å, respectively (Table 1 ▸, Figs. 2 ▸ *h* and 2 ▸ *l*). Coordinates have been deposited in the PDB (PDB entries 7njj and 7njg, respectively).

### 3.2. In-chip crystallization of a new protein variant   

Although the crystallization of model systems is an important quality control, we wanted to demonstrate that this method can also be used for novel structure determination of a previously unsolved protein. Moreover, we also sought to demonstrate that micro-crystallization can be achieved at protein concentrations typical for soluble proteins (∼5–25 mg ml^−1^).

#### 3.2.1. In-chip crystallization of human γS-crystallin   

To this end, we investigated an aggregation-prone, deamidation variant of the highly soluble human eye lens protein γS-crystallin. The deamidation variant of γS-crystallin used in this study is an extreme example of naturally occurring, age-related deamidation and is implicated in cataract formation (Wilmarth *et al.*, 2006 ▸; Lapko *et al.*, 2002 ▸). This variant has a total of nine substitutions of amide-containing amino acids to carboxylic acid-containing amino acids. To date, only two crystal structures have been reported for human γS-crystallin, presumably because these highly soluble crystallins resist crystallization, in keeping with their biological role (Thorn *et al.*, 2019 ▸; Purkiss *et al.*, 2002 ▸).

**Figure 4:**
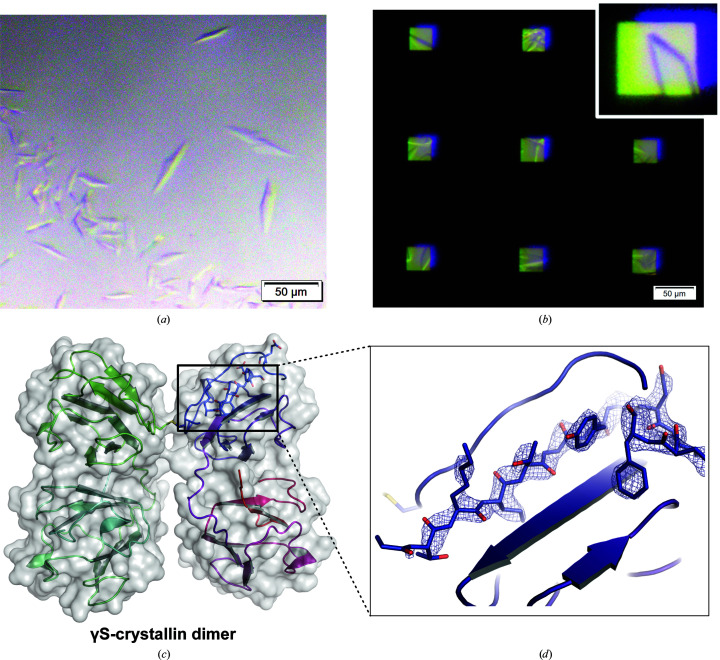
In-chip crystallization of γS-crystallin. Crystallization of the γS-crystallin variant using the same crystallization condition in traditional hanging drops (*a*) and in-chip crystallization (*b*). The inset shows an enlarged image of a crystal within the chip features. (*c*) The solved structure of the γS-crystallin variant with two monomers linked via a disulfide bond. (*d*) Representative section of the 2*F*_o_ − *F*_c_ electron-density map (contoured at 2σ) after refinement. Chain *A* is colored from blue to red from the N-terminus to the C-terminus, while chain *B* is colored from green to cyan.

Via conventional sparse-matrix screens, we identified a crystallization condition for this novel variant and then optimized it for higher nucleation rates. We found suitable hanging-drop vapor-diffusion crystallization to be achievable at a protein concentration of 11 mg ml^−1^ (Fig. 4 ▸ *a*). The same crystallization condition was then applied to the chip using the previously established protocol. In-chip crystal growth was observed in our custom crystallization trays after one day of incubation at 25°C (Fig. 4 ▸ *b*). Notably, the total amount of protein consumed to fill a chip was only 55 µg, demonstrating that this method is suitable to directly transfer hanging-drop vapor-diffusion crystallization conditions to the chip format without high sample consumption. This experiment also shows how standard high-throughput screening workflows can be utilized to enable serial crystallography with the HARE-chip technology. The structure was solved at a resolution of 3.0 Å from a single chip (Table 1 ▸, Figs. 4 ▸ *c* and 4 ▸ *d*). The refined structure has been deposited in the PDB (PDB entry 7nje).

#### 3.2.2. Structure of the novel γS-crystallin variant   

While Purkiss and coworkers determined the crystal structure of the human γS-crystallin truncated to just the C-terminal domain (PDB entry 1ha4; Purkiss *et al.*, 2002 ▸), Thorn and coworkers crystallized a dimer of γS-crystallin linked via an intermolecular disulfide bond between Cys24 in each monomer (PDB entry 6fd8; Thorn *et al.*, 2019 ▸). Solution-state NMR has also been employed in the study of human γS-crystallin; structures of the wild-type monomer (PDB entry 2m3t; Bru­baker & Martin, 2012 ▸; Kingsley *et al.*, 2013 ▸) and aggregation-prone single-site variants (PDB entries 2m3u and 6if9; Bari *et al.*, 2019 ▸; Kingsley *et al.*, 2013 ▸) have been solved. The γS-crystallin deamidation variant structure reported here crystallizes in space group *P*2_1_2_1_2_1_. It shows a disulfide-linked dimer at the same pair of cysteines (numbered Cys25 here) as the wild-type dimer structure PDB entry 6fd8. However, unlike PDB entry 6fd8, this novel structure arranges in a different orientation from the previously described QR configuration (Thorn *et al.*, 2019 ▸). Instead, the monomers are mirrored across the dimer interface (Fig. 4 ▸ *c*), with interfacial contacts arising from the disulfide bonds between Cys25 on each subunit and the δ-oxygen of Arg26 and τ-nitrogen of His87 on either subunit. The fold of the monomer units is highly similar to PDB entry 6fd8, with a global root-mean-square deviation (r.m.s.d.) of 0.463 Å derived from the alignment of chain *A* of PDB entry 6fd8 and chain *A* of the variant γS-crystallin dimer structure solved here (Thorn *et al.*, 2019 ▸). The mutated sites appear to play a significant role in the crystallization of this deamidated variant. Additional crystal-packing contacts are visible in this novel structure that are absent in other reported structures. Significantly, five of the nine sites of deamidation (Asp15, Glu121, Asp144, Glu17 and Glu107) create salt bridges to symmetry mates; these contacts are absent in the PDB entry 6fd8 and 1ha4 structures.

Deamidation and oxidation are frequently observed modifications in crystallins in cataractous lenses (Lampi *et al.*, 1998 ▸; Hains & Truscott, 2007 ▸, 2008 ▸; Wilmarth *et al.*, 2006 ▸). Deamidation has even been suggested to lead to increased disulfide-bond formation (Forsythe *et al.*, 2019 ▸; Vetter *et al.*, 2020 ▸). Human γS-crystallin, with its higher cysteine content compared with other lens crystallins, displays extensive deamidation and disulfide bonding in aged lenses (Skouri-Panet *et al.*, 2001 ▸; Hanson *et al.*, 1998 ▸; Ma *et al.*, 1998 ▸), and fulfills a complex role in the lens with the potential to mediate oxidative damage via disulfide exchange (Roskamp *et al.*, 2020 ▸). Although both deamidation and oxidation are suggested to result in minor structural changes, these small structural changes can still be critically detrimental to protein dynamics and stability (Forsythe *et al.*, 2019 ▸; Thorn *et al.*, 2019 ▸; Vetter *et al.*, 2020 ▸). Further work is being conducted to define the role of deamidation in the stability and aggregation propensity of γS-crystallin and to investigate the interplay of multiple post-translational modifications in cataract formation.

### 3.3. Intracellular protein crystallization   

Exploiting the intrinsic ability of cells to crystallize proteins represents an alternative approach to obtain protein microcrystals suitable for X-ray crystallography (Schönherr *et al.*, 2018 ▸). We have previously shown that serial femtosecond diffraction of micrometre-sized protein crystals directly in living cells on a fixed target (FT-SFX) enables high-resolution structure elucidation (Lahey-Rudolph *et al.*, 2021 ▸). The structure of HEX-1 from the fungus *N. crassa* crystallized in Sf9 insect cells was solved at 1.8 Å resolution using diffraction data from only a single chip collected within 12 min at the Linac Coherent Light Source (LCLS). Here, we used the same protein to test the applicability of fixed-target sample delivery for serial *in cellulo* diffraction data collection at a synchrotron source at room temperature.

HEX-1 is the Woronin body major protein in the filamentous fungus *N. crassa* and naturally forms crystals to seal the septal pores in the case of cell damage (Tenney *et al.*, 2000 ▸). Its spontaneous self-assembly into intracellular crystals also occurs in insect cells after infection by a recombinant baculovirus (rBV) encoding the HEX-1 gene. Regular, micrometre-sized hexagonal crystals grow reproducibly and with high efficiency in living insect cells (Lahey-Rudolph *et al.*, 2020 ▸). In the High Five insect cells used here, crystal growth is observed 48 h after rBV infection at the earliest, with a maximum of crystal-containing cells 96 h after infection. The cells produce spindle-like crystals with average dimensions of 26.3 ± 10.2 µm in length and 5.3 ± 1.5 µm in width, which are considerably larger than the crystal dimensions observed in Sf9 cells that we have used before (9.1 ± 3.2 µm in length and 3.5 ± 0.7 µm in width; Lahey-Rudolph *et al.*, 2021 ▸), but only marginally larger than the bottom openings of the chips that were used for the intracellular crystals (∼25 µm).

#### 3.3.1. Establishing HARE chips for *in cellulo* synchrotron diffraction data collection   

In a first approach, serial diffraction of the intracellular HEX-1 crystals was established using HARE chips on the P14.2 synchrotron beamline. High Five insect cells were infected with the HEX-1-encoding rBV in a six-well cell-culture plate using an MOI of 1. Intracellular crystal growth was confirmed by light microscopy four days after infection. Immediately before the diffraction experiment, the crystal-containing cells were stripped and 200 µl of cell suspension from a single well was loaded onto the chip using the previously described custom-made vacuum system (Mehrabi *et al.*, 2020 ▸). Serial diffraction data were collected directly without any additional steps. The diffraction details for HEX-1 intracellular crystals are listed in Table 1 ▸. Applying the parameters of a primitive hexagonal unit cell, 5520 of the recorded detector images were successfully indexed (a 27% indexing rate). The refined unit-cell parameters are comparable to those extracted from previous X-ray diffraction experiments (Yuan *et al.*, 2003 ▸; Lahey-Rudolph *et al.*, 2020 ▸, 2021 ▸), confirming the indicated similar composition of the *in vitro*-grown and *in cellulo*-grown HEX-1 crystals. The structural model of HEX-1 refined at 2.30 Å resolution is superimposable on that obtained from FT-SFX of *in cellulo*-grown HEX-1 (Lahey-Rudolph *et al.*, 2021 ▸), with an overall r.m.s.d. of 0.225 Å for 141 C^α^ atoms.

#### 3.3.2. Structure determination using *in situ*-crystallized HEX-1   

**Figure 5:**
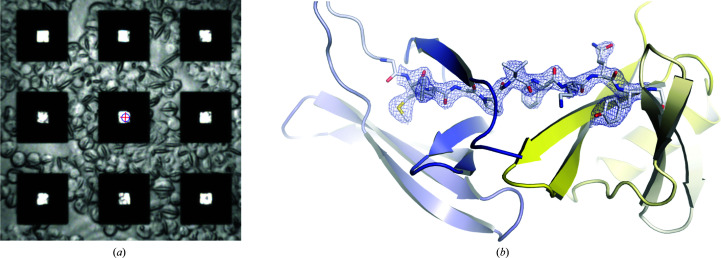
*In situ* crystallization of intracellular HEX-1 crystals. (*a*) Fungal protein HEX-1 crystallized *in cellulo* on the surface and in the features of the chip. (*b*) The HEX-1 structure with the N-terminal domain shown in blue and the C-terminal domain shown in yellow with a representative section of the 2*F*_o_ − *F*_c_ electron-density map (contoured at 2σ) after refinement from the chip 1 (*in situ* data set).

In a second approach, *in situ* crystallization of HEX-1 was tested in living High Five cells that had been loaded into the features of the HARE chips. After adhesion onto the poly-d-lysine-coated silicon chip, the High Five cells were infected with the HEX-1-encoding rBV and cultured on the chip surface until the diffraction experiment (96 h post-infection). Almost complete coverage of the chip surface by the cell layer was detected by light microscopy, clearly extending into the features (Fig. 5 ▸ *a*). Spindle-shaped crystals were observed in the majority of cells, exhibiting a comparable morphology and dimensions to those of intracellular HEX-1 crystals grown in a cell-culture plate. For serial diffraction data collection, the chip was removed from the well of the cell-culture plate and the excess culture medium was removed before mounting. The diffraction details for HEX-1 *in situ* crystals are comparable to those obtained after loading HEX-1 crystal-containing cells onto the chip (Table 1 ▸). Although the indexing rate was slightly decreased (8%), the diffraction data from a single chip enabled structure determination at a resolution of 2.50 Å (Fig. 5 ▸ *b*). The slightly different resolution depends on the number of indexed patterns and thus on the multiplicity. While 5520 patterns were successfully indexed after diffraction of the loaded crystal-containing cells (PDB entry 7nji), resulting in a multiplicity of 93, only 2111 patterns were indexed after diffraction of *in situ*-crystallized HEX-1 (PDB entry 7njh), resulting in a multiplicity of 37. The refined HEX-1 structures solved by *in cellulo* diffraction of loaded crystal-containing cells and of *in situ*-grown crystals have been deposited in the Protein Data Bank (PDB entries 7nji and 7njh, respectively).

## 4. Discussion   

As in any crystallographic project, the availability of homogenous, well diffracting microcrystals is an experimental bottleneck in fixed-target serial crystallography. Therefore, previous studies have focused on reducing sample consumption and crystal handling of batch-crystallized solutions. Lyubimov and coworkers loaded microfluidic trap arrays with only 5 µl of crystal slurry (Lyubimov *et al.*, 2015 ▸), while Murray and coworkers transferred even smaller volumes (<2 µl) of microcrystal suspensions onto X-ray-transparent silicon nitride chips (Murray *et al.*, 2015 ▸). Recently, Davy and coworkers used acoustic droplet ejection to load ‘Oxford photo-chips’ (which are highly similar to the HARE chips) with only 3 µl of crystal slurry grown in batch (Davy *et al.*, 2019 ▸). Although these methods provide a remarkable sample reduction, they all rely on pre-crystallized material. In contrast, crystallization on the surface of fixed-target supports has also been described before.

While Ren and coworkers used monocrystalline quartz plates as a support structure for on-chip crystallization of various sizes (Ren *et al.*, 2018 ▸), Lee and coworkers developed a one-dimensional fixed-target system in which microcrystals are loaded into an array of polyimide tubes. This method allows low sample consumption and was shown to successfully grow crystals *in situ* via batch crystallization. However, unlike the HARE chips, these techniques would not allow the ligand-mixing studies that have become important for reaction initiation in time-resolved experiments, and the method of Lee and coworkers does not support vapor-diffusion-driven *in situ* crystallization (Lee *et al.*, 2020 ▸). In contrast, Lieske and coworkers developed an on-chip vapor-diffusion crystallization method on the surface of the porous silicon ‘Roadrunner’ chips that were also used for ligand soaking (Lieske *et al.*, 2019 ▸). In the methods mentioned above the crystals are randomly positioned rather than in predetermined locations, preventing the use of the HARE method for time-resolved crystallography (Schulz *et al.*, 2018 ▸). Opara and coworkers developed an *in situ* crystallization method using silicon nitride membranes that is highly similar to the approach described here, as the crystals are located in predefined positions and are crystallized using a vapor-diffusion approach (Opara *et al.*, 2017 ▸). In contrast to the HARE chips, the silicon nitride membranes are closed on one side of the chip, which may lead to different crystallization results. In general, an exact comparison of the different approaches described above is complicated by different form factors and numbers of features as well as the concentrations of the crystal slurries or protein solutions.

As a practical advantage, crystallization within HARE chips requires minimal equipment and can thus be carried out in nonspecialized laboratories. With the exception of the HARE chips, the required materials are common laboratory supplies or can be substituted with similar tools. The crystallization trays are readily accessible through 3D printing and the files are available with this manuscript. At the same time, this in-chip crystallization approach can be used to seamlessly extend existing high-throughput crystallization workflows. The major benefit of this approach is to directly transfer optimized crystallization conditions from droplets to the chip format, without or with limited additional adaptation. Notably, optimization does not require the use of an entire chip, but partial chip screening can be used to optimize the size and density of crystals using <1 µl protein solution in a single column of the HARE-chip compartments. Importantly, as the crystals are confined to grow inside the chip features and only a limited supply of protein is available within each feature, a crystal size limit compatible with time-resolved applications is inherent in the crystallization setup. The feasibility of this workflow is not only demonstrated by the model systems, but also by the successful structure determination of a novel γS-crystallin variant, a group of proteins that are known to be rather recalcitrant towards crystallization. Thus, for novel systems, promising conditions for in-chip crystallization can be found using established, conventional screening approaches, which can subsequently be extended to in-chip crystallization.

The reported model protein structures were solved using data from a single chip loaded with approximately 140–400 µg of protein for lysozyme, proteinase K and xylose isomerase, which is slightly less than that consumed by loading crystal slurries via vacuum loading (Mehrabi, Schulz, Agthe *et al.*, 2019 ▸). The median hit rate of 25% indicates that a single chip is usually sufficient for structure determination (Mehrabi *et al.*, 2021 ▸). An interesting observation is that this can be substantially increased by a simple short centrifugation step, an aspect that needs further detailed exploration in future experiments. However, in-chip crystallization is not limited to model systems. The γS-crystallin structure demonstrates that in-chip crystallization can also be applied to nonmodel proteins. This structure also emphasizes that the typical protein concentrations (here 11 mg ml^−1^) commonly found in vapor-diffusion crystallization screens are amenable to HARE-chip crystallization. At this protein concentration the overall sample consumption reduces to 55 µg protein per chip. This is a five–tenfold reduction compared with the amount of protein typically used in vacuum loading of crystal slurries using the HARE chip. However, in comparison to the model systems, γS-crystallin showed rather low hit rates (6%), and it is likely that its comparably low protein concentration affected the final crystal density. Presumably, further optimization of the nucleation propensity, or the use of seed stocks, would have a positive effect on the crystallization and lead to higher in-chip hit rates (Beale *et al.*, 2019 ▸). While the hit rate determines the efficiency of the data-collection experiment, serial crystallo­graphy also permits an estimation of the efficiency of the crystallization itself. To this end, the number of diffraction patterns can be compared with the total amount of protein. While the model systems showed a median number of 21 diffraction patterns per microgram of protein, γS-crystallin showed 27 diffraction patterns per microgram of protein and thus a comparable efficiency of crystallization. This emphasizes the potential of HARE-chip crystallization to reduce the amount of protein that is required for serial crystallography. We note that automation of these workflows in the future will likely lead to a further reduction of the sample requirements.

In addition to reduced sample consumption, another advantage of in-chip crystallization is the reduced crystal handling compared with the vacuum-loading method previously used in HARE time-resolved studies. With in-chip crystallization, the crystals can remain unperturbed after growth, with no transfer steps between growth and data collection. This could be particularly helpful in intracellular crystallization as these samples are often highly sensitive, especially when the crystals are purified from the cells. However, intracellular protein crystallization offers some distinct advantages over traditional screening approaches: crystals can be grown in a cellular environment with natural post-translational modifications (Redecke *et al.*, 2013 ▸) alongside a diverse array of small molecules and possible cofactors to support crystallization; indeed, cofactors have been discovered via this method (Nass *et al.*, 2020 ▸). *In cellulo* crystallization is also more scalable and eschews laborious purification steps. We have recently shown that silicon chips are ideally suited for serial crystallography, providing high-resolution diffraction of HEX-1 crystals within intact cells via serial femtosecond crystallography (SFX) at an XFEL (Lahey-Rudolph *et al.*, 2021 ▸). Here, we confirm that fixed-target delivery of crystal-containing cells also enables fast and efficient data collection via serial synchrotron crystallography (SSX) at room temperature. It is possible that the resolution of the diffraction data is limited by the reduced photon flux of the synchrotron source. However, the HEX-1 structure determined via SSX is directly superimposable with that determined via fixed-target SFX, validating the comparability of SSX and SFX data (Mehrabi *et al.*, 2021 ▸).

Moreover, insect cells can be cultivated and infected directly on the surface of the HARE chips without affecting the growth and the quality of the HEX-1 crystals. The *in situ* approach provides the advantage of avoiding any cell-transfer procedures; thus, the sample remains unperturbed until X-ray exposure. In contrast, loading cells containing preformed crystals requires the removal of excess medium. If harsh vacuum-loading systems are used, this could affect the cell integrity and thus the crystal quality, since virus-infected cells are particularly sensitive to mechanical stress. The viral infection might also inhibit the attachment of the crystal-containing cells to the chip surface after loading, leading to the loss of some cells during removal of the cell-culture medium and thus to reduced hit rates. These limitations should be overcome by the *in situ* approach, but the indexing rate did not improve. This is attributed to problems in growing the cell monolayer up to the center of the chip features, where any support is missing. Thus, some features did not contain a crystal-containing cell in the volume that is hit by the X-ray beam. It needs to be tested in future studies whether a very gentle centrifugation centering will improve the hit rates, as observed for other samples.

In addition to the advantage of exploiting intracellular crystals via SSX, this work demonstrates that the main advantage of HARE-chip delivery of intracellular crystals is a significant reduction in the required material. Per indexed diffraction pattern, this method has an almost 2000-fold lower sample consumption compared with liquid-jet SFX approaches previously performed to solve the structures of *T. brucei* CatB and IMPDH (Redecke *et al.*, 2013 ▸; Nass *et al.*, 2020 ▸).

## 5. Conclusions   

Here, we present a new crystallization technique inside HARE chips that can substantially reduce protein consumption and sample handling for serial X-ray crystallography. Canonical vapor-diffusion crystallization conditions can be directly transferred to crystallization inside the HARE chips. The direct growth of protein microcrystals within precisely defined features is therefore compatible with the HARE method for efficient time-resolved crystallography. For systems that are costly to produce, resist batch crystallization, form highly delicate crystals or crystallize in living cells, in-chip crystallization may offer distinct advantages over other sample-preparation techniques.

## Supplementary Material

3D-Files to re-generate the HARE-chip crystallization tray. DOI: 10.1107/S2059798321003855/nj5304sup1.txt

Crystal hit-maps. DOI: 10.1107/S2059798321003855/nj5304sup2.pdf

PDB reference: in-chip-grown HEX-1, 7njh

PDB reference: vacuum-loaded HEX-1, 7nji

PDB reference: in-chip-crystallized lysozyme, 7nkf

PDB reference: in-chip-crystallized lysozyme, with centrifugation, 7njf

PDB reference: in-chip-crystallized xylose isomerase, 7njg

PDB reference: in-chip-crystallized proteinase K, 7njj

PDB reference: in-chip-crystallized γS-crystallin mutant, 7nje

## Figures and Tables

**Table:** Values in parentheses are for the highest resolution shell.

	Lysozyme				Proteinase K		
	Chip 1	Chip 2	Chip 3	Chip 4	Chip 1	Chip 2	Chip 3
PDB code	—	—	7nkf	7njf	—	—	7njj
Crystal centering	—	—	—	Yes	—	—	—
Normalized protein used (µg)	138.9	263.3	325.0	325.0	243.0	243.0	240.7
Collected features	10944	20736	25600	25600	20736	20736	20543
Indexed features	2938	3707	7498	14840	4574	2221	7618
Indexed diffraction patterns	2991	3973	8611	20911	5294	2502	10489
Indexing rate† (%)	27	19	34	82	26	12	51
Indexed patterns per microgram of protein	22	15	26	64	22	10	44
Space group	*P*4_3_2_1_2	*P*4_3_2_1_2	*P*4_3_2_1_2	*P*4_3_2_1_2	*P*4_3_2_1_2	*P*4_3_2_1_2	*P*4_3_2_1_2
*a*, *b*, *c* (Å)	78.6, 78.6, 38.6	78.5, 78.5, 38.8	78.8, 78.8, 38.7	78.5, 78.5, 38.8	68.3, 68.3, 108.2	68.5, 68.5, 108.6	68.5, 68.5, 108.7
α, β, γ (°)	90, 90, 90	90, 90, 90	90, 90, 90	90, 90, 90	90, 90, 90	90, 90, 90	90, 90, 90
Resolution (Å)	78.74–1.70 (1.76–1.70)	78.74–1.70 (1.76–1.70)	55.87–1.70 (1.76–1.70)	55.56–1.70 (1.76–1.70)	108.70–1.65 (1.71–1.65)	108.70–1.65 (1.71–1.65)	68.49–1.65 (1.71–1.65)
CC* (%)	98.3 (93.3)	98.7 (91.0)	98.8 (97.2)	99.6 (98.2)	95.0 (88.0)	85.8 (74.2)	97.3 (76.3)
*R*_split_ (%)	19.69 (46.63)	17.67 (55.87)	16.55 (22.52)	10.0 (21.5)	33.23 (48.24)	57.24 (81.11)	26.03 (67.77)
〈*I*/σ(*I*)〉	5.1 (2.1)	5.2 (1.8)	6.8 (4.7)	10.1 (4.6)	3.7 (2.4)	2.4 (1.5)	3.9 (1.7)
Multiplicity	68 (40)	81 (48)	174 (105)	408 (248)	37 (20)	17 (9)	72 (39)
Completeness (%)	100.00 (100.00)	100.00 (100.00)	100.00 (100.00)	100.00 (100.00)	99.98 (99.97)	99.82 (99.23)	100.00 (100.00)
Resolution range for refinement (Å)			55.72–1.70 (1.83–1.70)	55.51–1.70 (1.76–1.70)			57.95–1.65 (1.71–1.65)
No. of reflections			13920 (1358)	13833 (1356)			31851 (3100)
*R*_work_ (%)			17.50 (19.64)	17.40 (21.30)			17.79 (25.34)
*R*_free_ (%)			21.18 (22.33)	20.28 (27.94)			20.33 (28.75)
Mean *B* factor (Å^2^)			22.49	24.6			15.29
No. of non-H atoms							
Protein			1023	1013			2031
Ligands/ions			1	1			4
Water			74	69			209
R.m.s. deviations							
Bond lengths (Å)			0.013	0.006			0.006
Bond angles (°)			1.26	0.93			0.77
Ramachandran statistics							
Favored (%)			99.21	98.41			97.11
Allowed (%)			0.79	1.59			2.89
Outliers (%)			0.00	0.00			0.00

**Table:** 

	Proteinase K				HEX-1	
	Chip 4	Chip 5	Xylose isomerase	γS-crystallin mutant	Loaded	*In situ*
PDB code	—	—	7njg	7nje	7nji	7njh
Crystal centering	—	Yes	Yes	Yes	—	—
Normalized protein used (µg)	300.0	202.5	400.0	55.0	—	—
Collected features	25600	17280	25600	25600	20736	25281
Indexed features	2751	3661	3040	1482	5100	2023
Indexed diffraction patterns	3855	4360	5186	1510	5520	2111
Indexing rate† (%)	15	25	20	6	27	8
Indexed patterns per microgram of protein	13	22	13	27		
Space group	*P*4_3_2_1_2	*P*4_3_2_1_2	*I*222	*P*2_1_2_1_2_1_	*P*6_5_22	*P*6_5_22
*a*, *b*, *c* (Å)	68.4, 68.4, 108.4	68.4, 68.4, 108.2	94.5, 103.2, 99.8	38.4, 89.2, 111.3	58.9, 58.9, 193.0	58.6, 58.6, 192.7
α, β, γ (°)	90, 90, 90	90, 90, 90	90, 90, 90	90, 90, 90	90, 90, 120	90, 90, 120
Resolution (Å)	68.49–1.65 (1.71–1.65)	68.49–1.65 (1.71–1.65)	71.94–1.90 (1.97–1.90)	69.44–3.00 (3.11–3.00)	192.31–2.30 (2.38–2.30)	64.10–2.50 (2.59–2.50)
CC* (%)	92.5 (79.1)	95.4 (78.3)	93.9 (76.6)	98.9 (88.5)	97.6 (74.3)	96.0 (74.8)
*R*_split_ (%)	41.50 (66.25)	32.91 (62.75)	41.28 (96.66)	28.96 (110.43)	25.00 (100.71)	28.39 (120.53)
〈*I*/σ(*I*)〉	2.9 (1.8)	3.6 (1.9)	2.3 (1.1)	2.5 (1.0)	3.2 (1.2)	3.1 (0.9)
Multiplicity	45 (25)	45 (24)	111 (77)	48 (34)	93 (65)	38 (26)
Completeness (%)	100.00 (99.97)	99.99 (99.97)	100.00 (100.00)	99.96 (100.00)	100.00 (100.00)	99.87 (99.86)
Resolution range for refinement (Å)			51.57–1.90 (1.97–1.90)	69.60–3.00 (3.11–3.00)	51.01–2.30 (2.38–2.30)	50.71–2.50 (2.59–2.50)
No. of reflections			38717 (3834)	8132 (787)	9522 (917)	7394 (706)
*R*_work_ (%)			20.16 (27.89)	23.50 (37.96)	21.93 (31.38)	22.02 (34.15)
*R*_free_ (%)			24.40 (32.25)	27.11 (47.85)	26.01 (37.53)	25.53 (36.60)
Mean *B* factor (Å^2^)			22.74	52.64	47.52	55.55
No. of non-H atoms						
Protein			3076	2903	1128	1128
Ligands/ions			1	0	0	0
Water			265	16	28	13
R.m.s. deviations						
Bond lengths (Å)			0.003	0.004	0.01	0.007
Bond angles (°)			0.6	0.84	1.16	0.89
Ramachandran statistics						
Favored (%)			96.62	95.93	95.17	95.17
Allowed (%)			3.12	3.49	3.45	2.76
Outliers (%)			0.26	0.58	1.38	2.07

† The indexing rate describes the indexed diffraction patterns per number of collected images.
