# Supplementary material for: A simple vapor-diffusion method enables protein crystallization inside the HARE serial crystallography chip
Source: Acta Crystallogr D Struct Biol. 2021 May 19;77(Pt 6):820–34. doi: 10.1107/S2059798321003855 (PMC8171066; doi:10.1107/S2059798321003855)

STRUCTURAL
BIOLOGY

Volume 77 (2021)

Supporting information for article:

A simple vapor-diffusion method enables protein crystallization inside the HARE serial crystallography chip

Brenna Norton-Baker, Pedram Mehrabi, Juliane Boger, Robert Schönherr, David von Stetten, Hendrik Schikora, Ashley O. Kwok, Rachel W. Martin, R. J. Dwayne Miller, Lars Redecke and Eike C. Schulz

A simple vapor diffusion method enables protein crystallization inside the HARE serial crystallography chip

Brenna Norton-Baker[1,2], Pedram Mehrabi[1,3], Juliane Boger[4], Robert Schönherr[4,5], David von Stetten[6], Hendrik Schikora[7], Ashley O. Kwok[2], Rachel W. Martin[2,8], R.J. Dwayne Miller[9,10], Lars Redecke[4,5] and Eike C. Schulz[1,3]*

[1]Department for Atomically Resolved Dynamics, Max-Planck-Institute for Structure and Dynamics of Matter, Luruper Chaussee 149, 22761 Hamburg, Germany
[2]Department of Chemistry, University of California, Irvine 92697-2025, United States
[3]Hamburg Centre for Ultrafast Imaging, Universität Hamburg, HARBOR, Luruper Chaussee 149, 22761 Hamburg, Germany
[4]Institute of Biochemistry, Center for Structural and Cell Biology in Medicine, University of Lübeck, Ratzeburger Allee 160, D-23562 Lübeck, Germany
[5]Deutsches Elektronen-Synchrotron (DESY), Photon Science, Notkestrasse 85, D-22607 Hamburg, Germany
[6]European Molecular Biology Laboratory, Hamburg Unit c/o Deutsches Elektronen-Synchrotron, 22607 Hamburg, Germany
[7]Scientific Support Unit Machine Physics, Max-Planck-Institute for Structure and Dynamics of Matter, Luruper Chaussee 149, 22761 Hamburg, Germany
[8]Department of Molecular Biology and Biochemistry, University of California, Irvine 92697-3900, United States
[9]Department of Physics, Universität Hamburg, Jungiusstrasse 9, 20355 Hamburg, Germany
[10]Departments of Chemistry and Physics, University of Toronto, 80 St. George Street, Toronto, Ontario, M5S 3H6, Canada

*to whom correspondence should be addressed:
eike.schulz@mpsd.mpg.de

Supplementary Material

Supplementary Figures

a) Hit-map: a blue dot on the grey outline of the chip compartments indicates a feature for which one or more protein diffraction pattern(s) could be obtained. For partially collected chips, the missing parts are indicated in yellow.

b) Resolution histogram of the diffraction patterns obtained for each chip.

c) Unit cell parameter histogram of the diffraction patterns obtained for each chip.

Lysozyme

Chip1

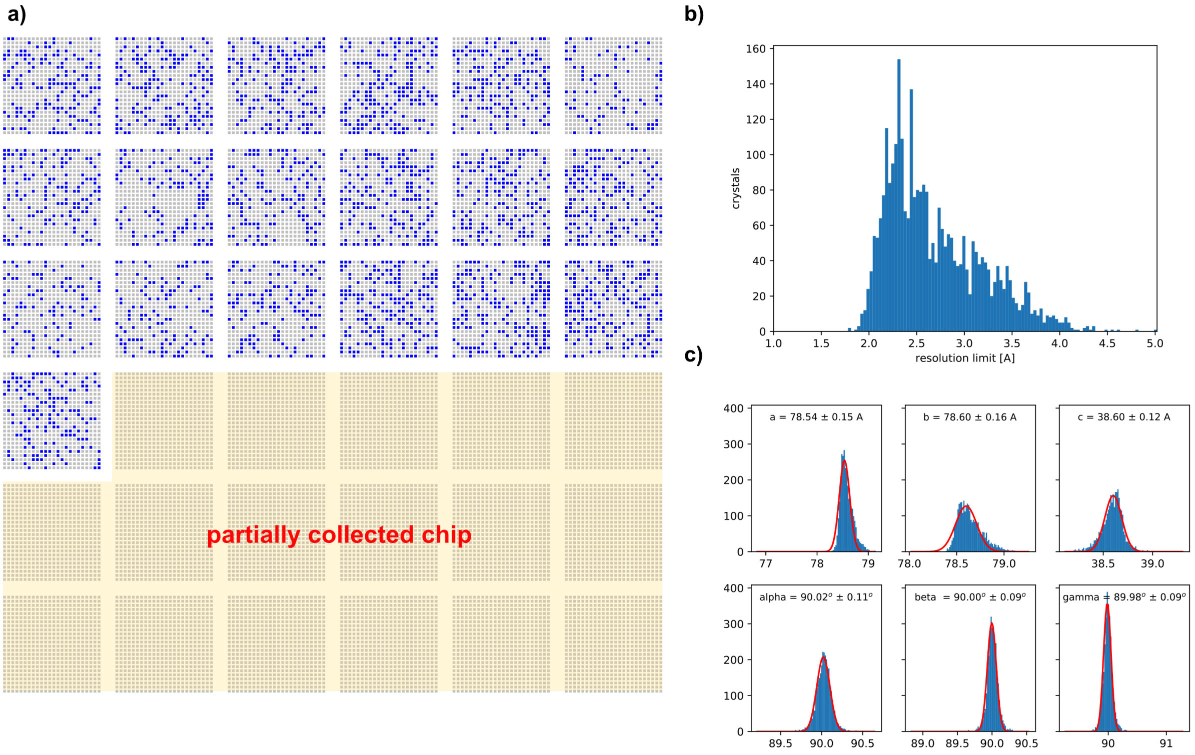

Chip2

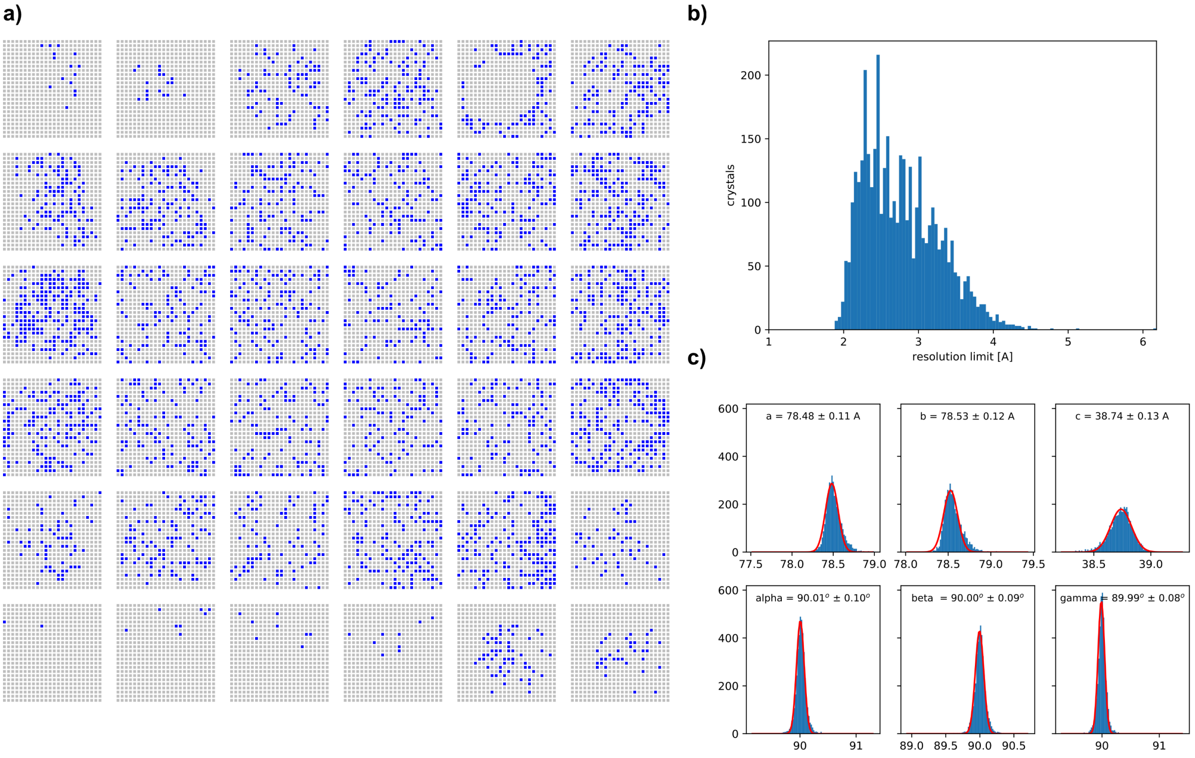

Chip3

a)

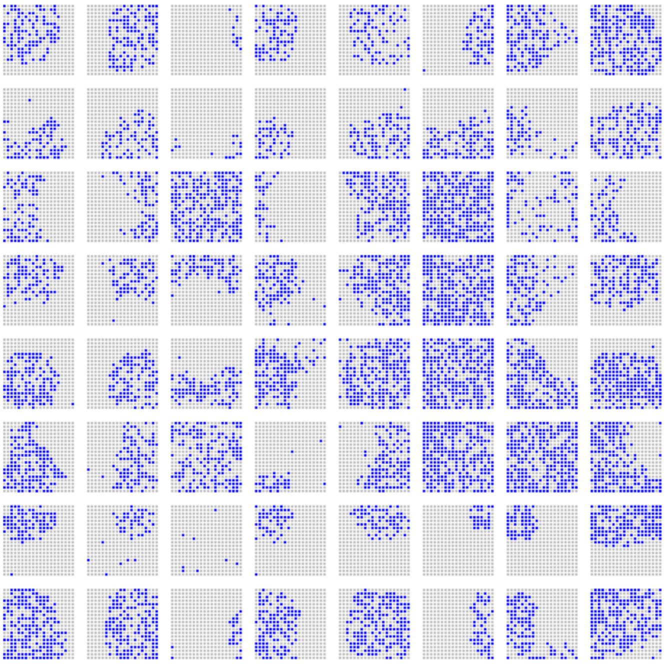

b)

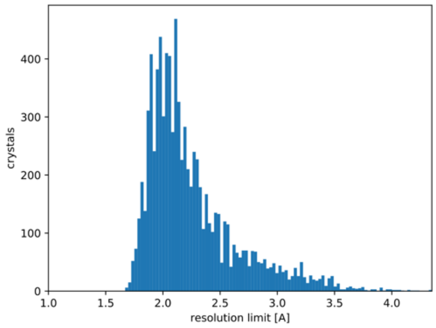

c)

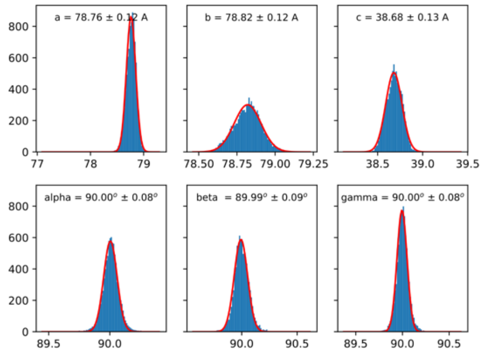

Chip4

a)

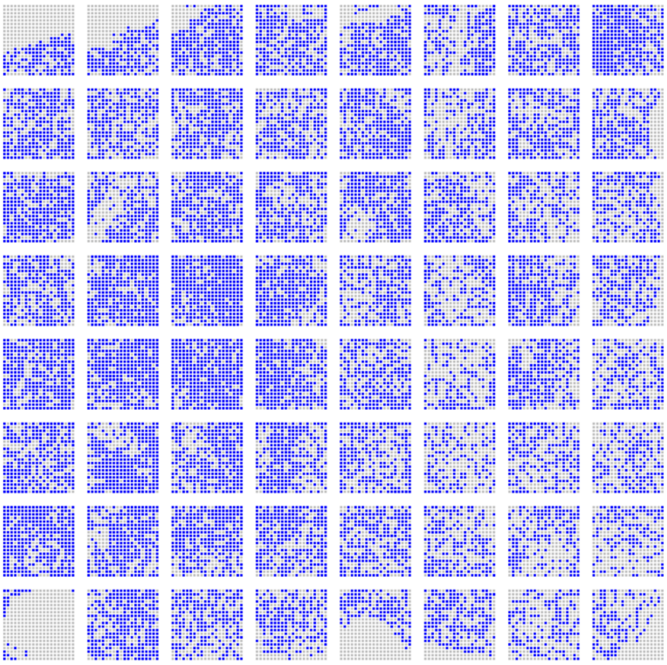

b)

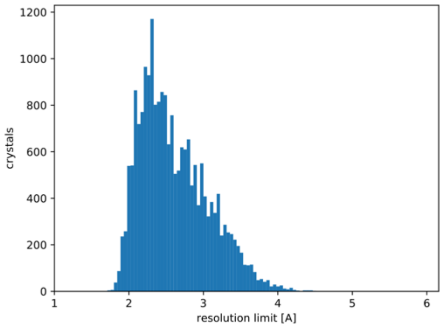

c)

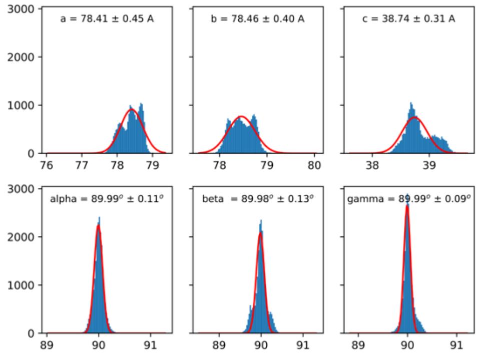

Proteinase K

Chip1

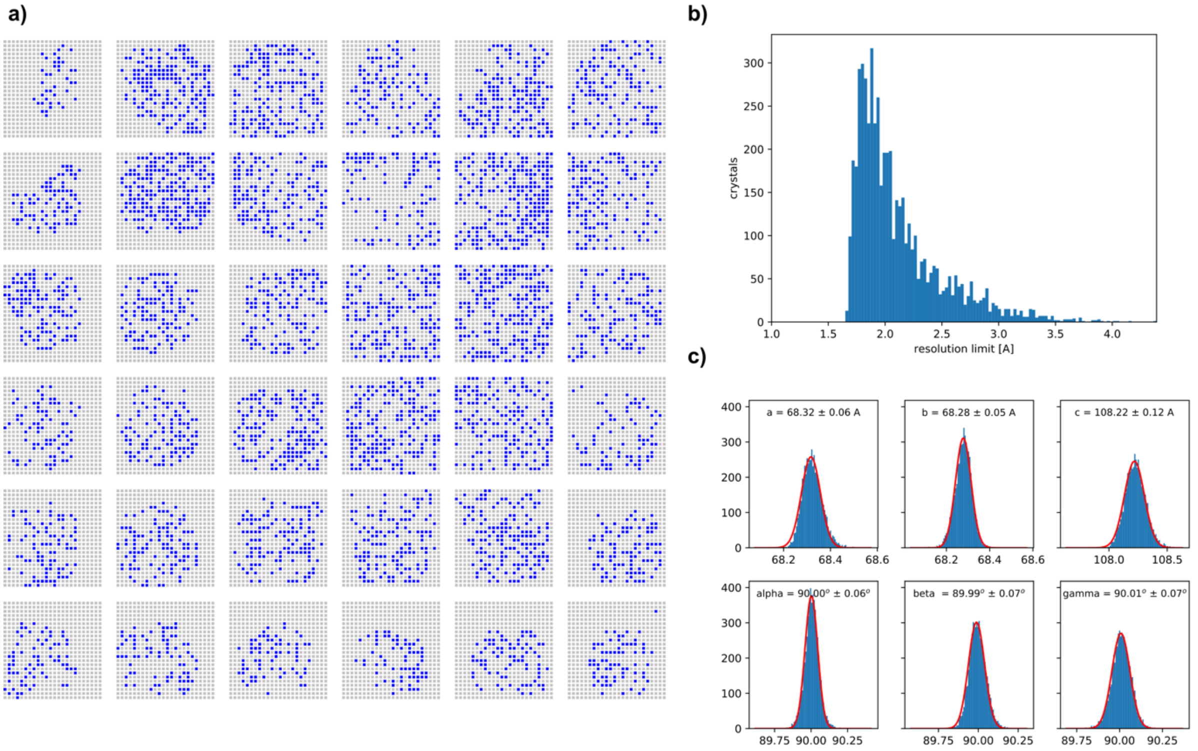

Chip2

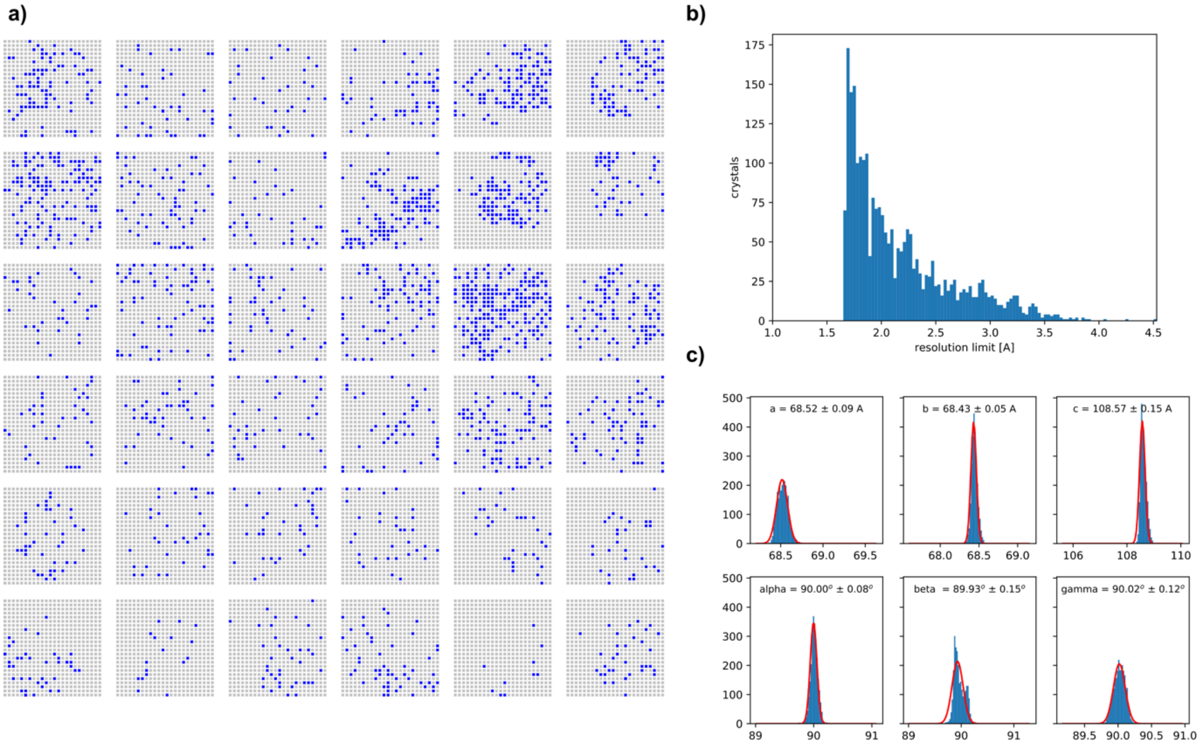

Chip3

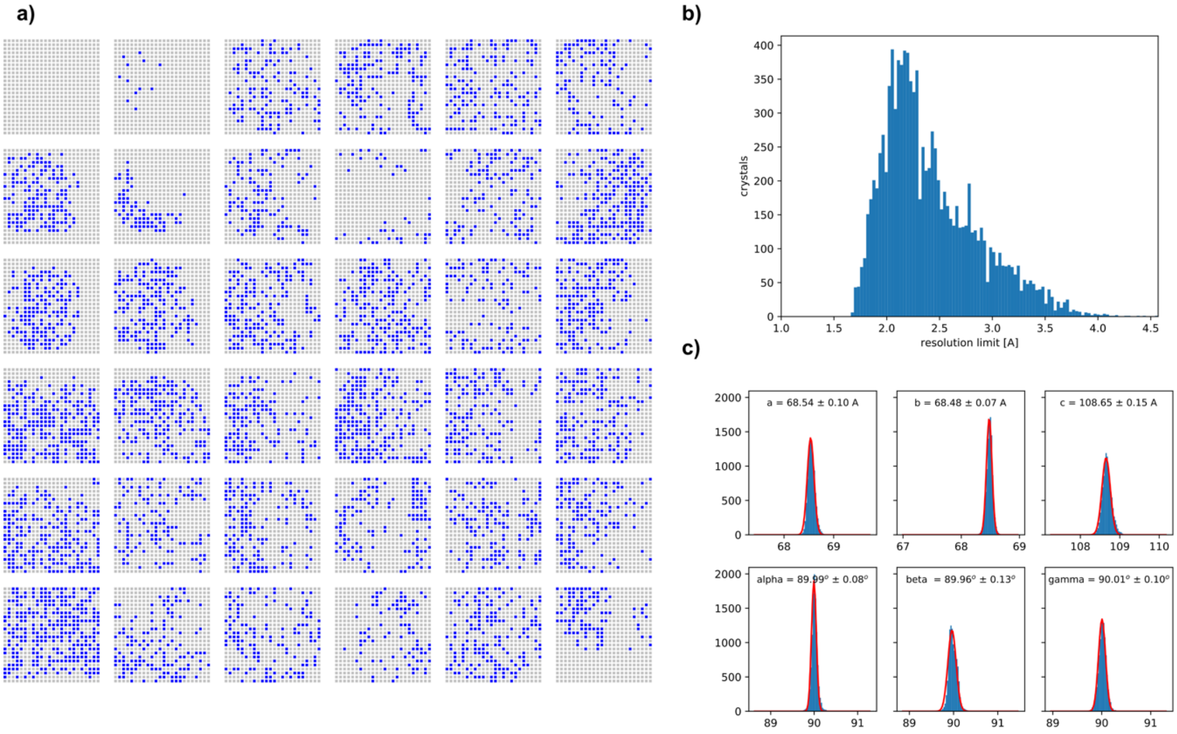
a)
b)
c)
crystals
resolution limit [A]
a = 68.54 ± 0.10 A
b = 68.48 ± 0.07 A
c = 108.65 ± 0.15 A
alpha = 89.99° ± 0.08°
beta = 89.96° ± 0.13°
gamma = 90.01° ± 0.10°

Chip4

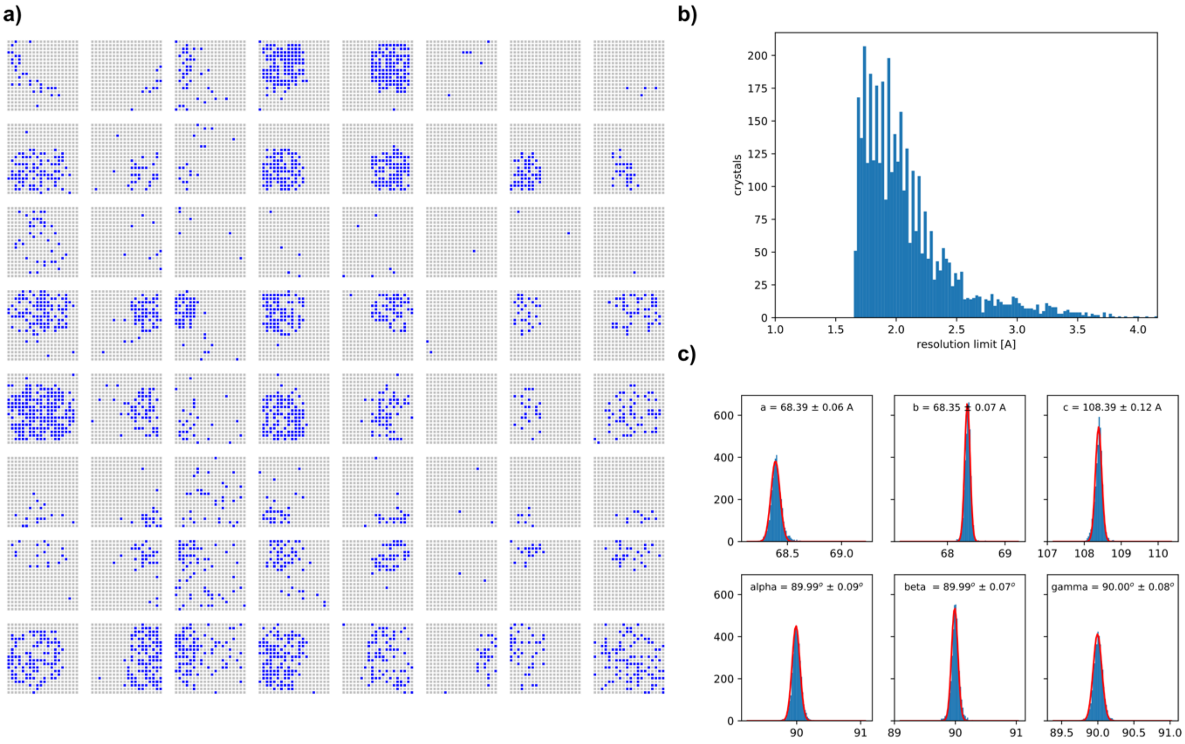
a)
b)
c)
crystals
resolution limit [A]
a = 68.39 ± 0.06 A
b = 68.35 ± 0.07 A
c = 108.39 ± 0.12 A
alpha = 89.99° ± 0.09°
beta = 89.99° ± 0.07°
gamma = 90.00° ± 0.08°

Chip5

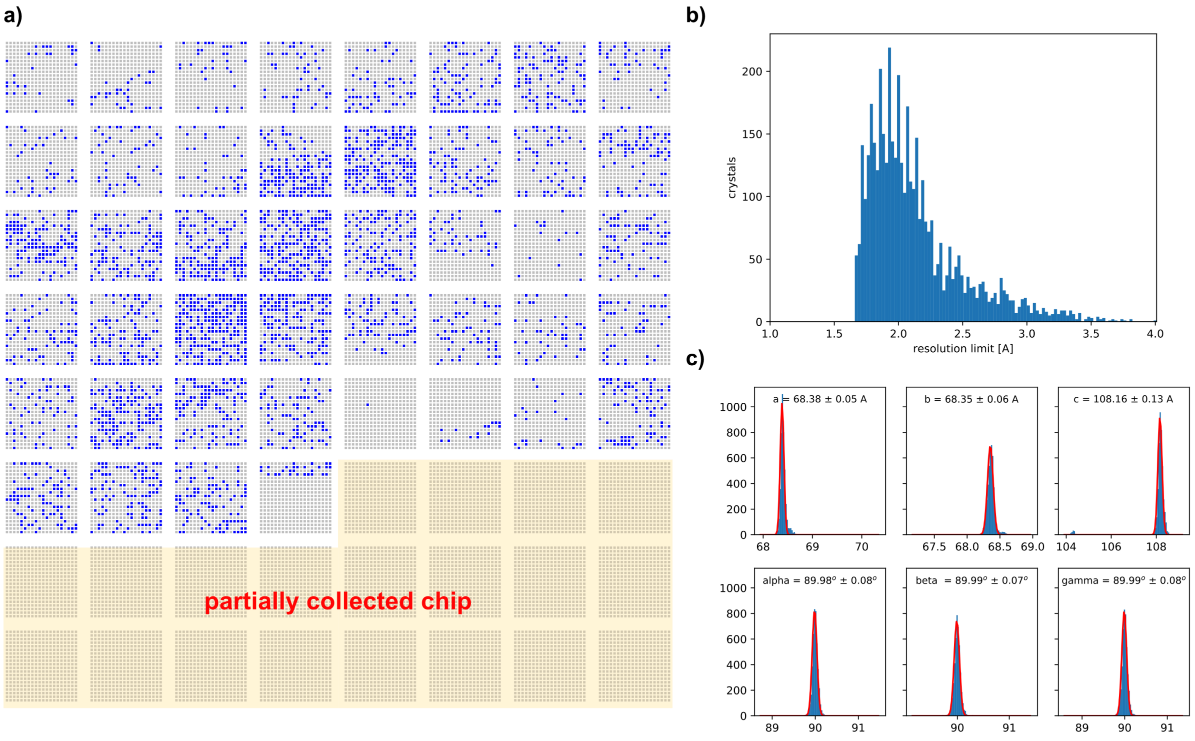

Xylose isomerase

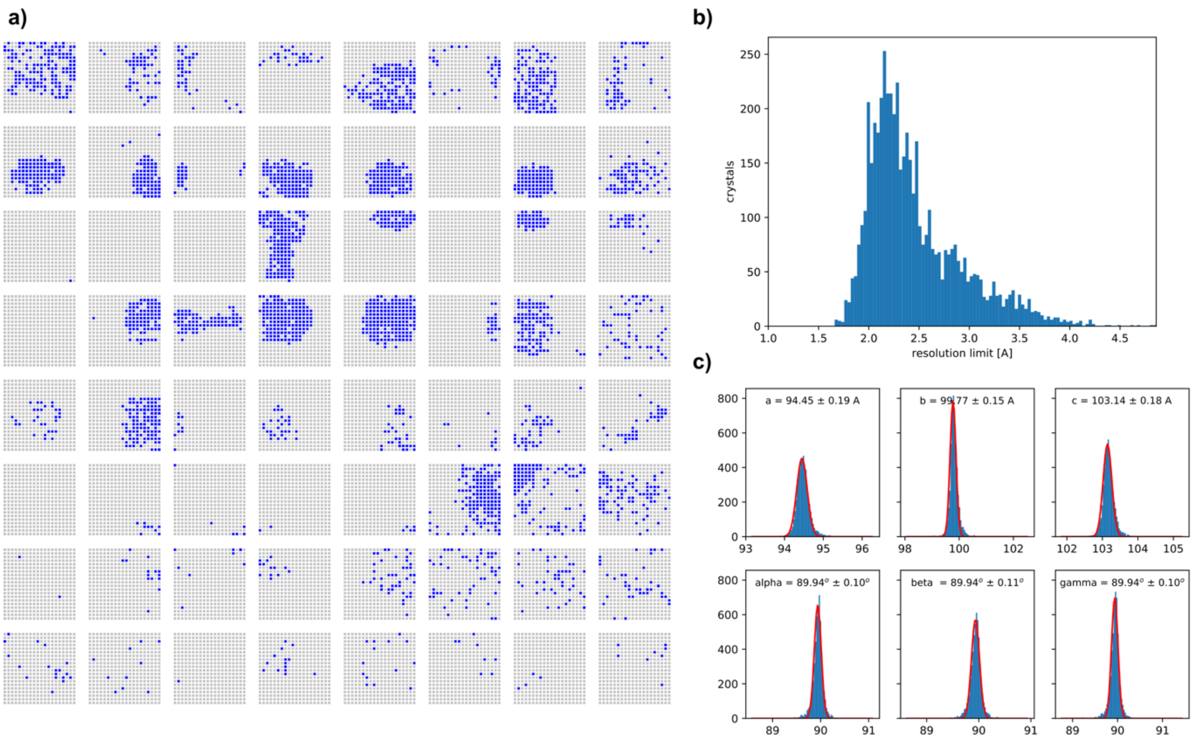

γS-crystallin mutant

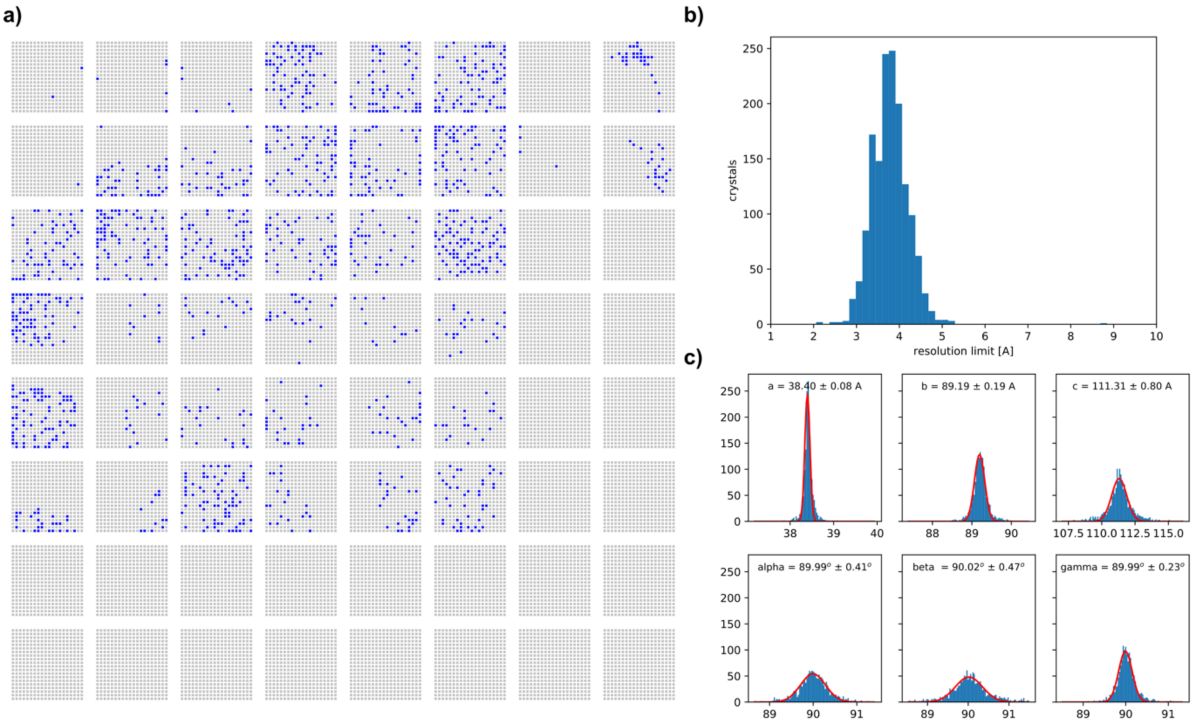

Hex-1

Chip1 (loaded)

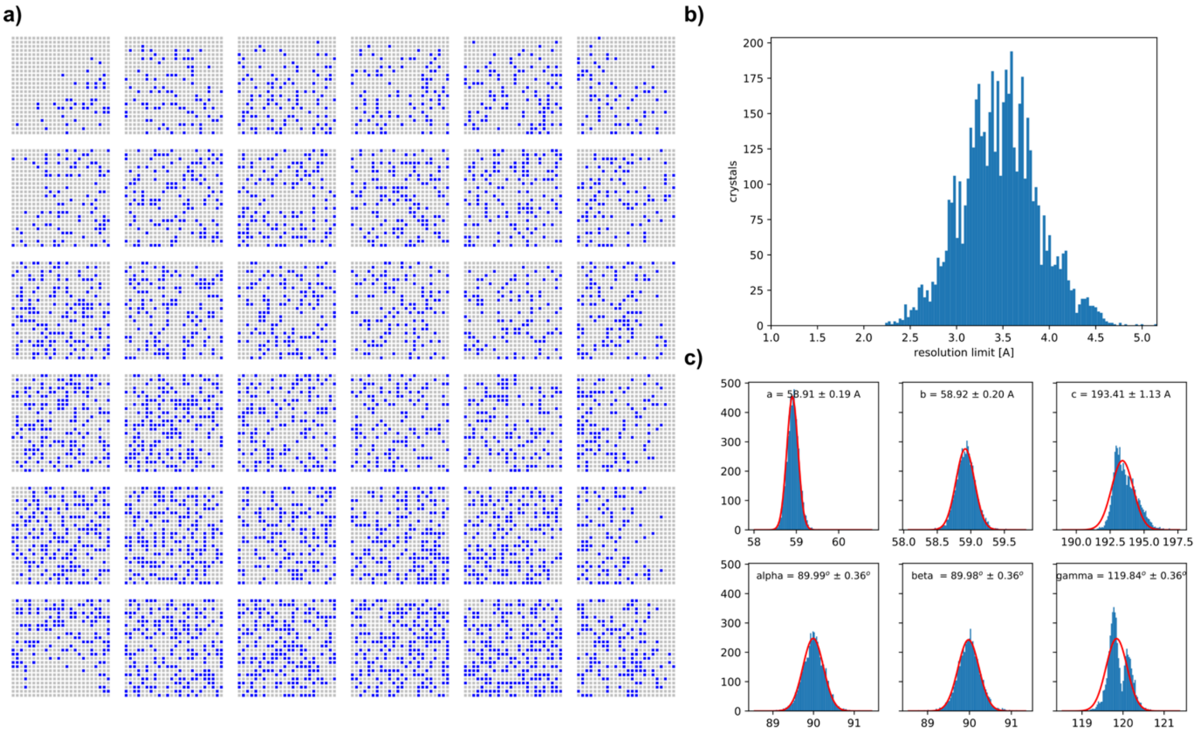

Chip2 (in-situ)

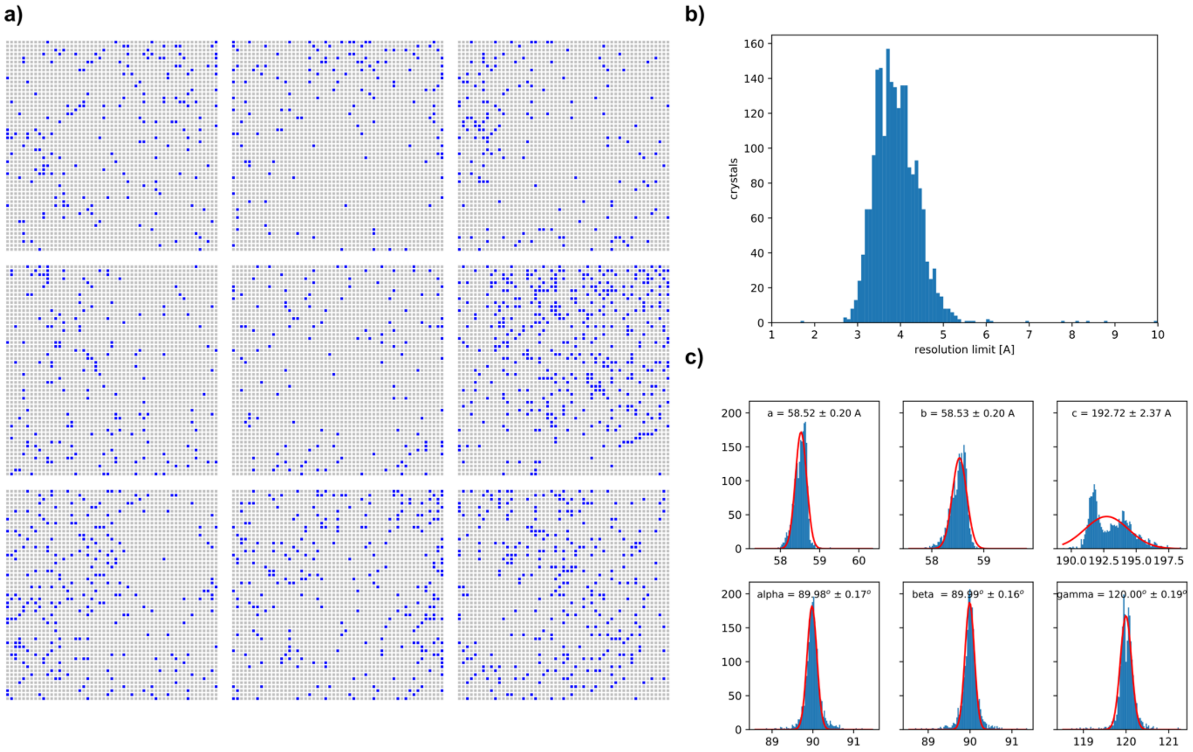

Supplement: Supplementary file 2 [file d-77-00820-sup2.pdf]
